# Construction and validation of multiple machine learning models for influencing factors of postpartum post-traumatic stress disorder in primiparas

**DOI:** 10.3389/fpsyt.2026.1838321

**Published:** 2026-07-15

**Authors:** Li Guo, Yiju Sun

**Affiliations:** Hefei Maternal and Child Health Hospital, Hefei, China

**Keywords:** gradient boosting, IFPHRM, machine learning, postpartum post-traumatic stress disorder, primiparas, risk prediction

## Abstract

**Objective:**

To analyze the multidimensional factors associated with postpartum post-traumatic stress disorder (PP-PTSD) in primiparas based on the Integrated Framework for Population Health Risk Management (IFPHRM), multiple machine learning-based predictive models were constructed and externally validated to identify high-risk individuals and to provide a robust evidence base for targeted preventive interventions.

**Methods:**

This cross-sectional study consecutively enrolled 1, 135 primiparous women from the Department of Obstetrics at Hefei Maternal and Child Health Hospital between June 2024 and May 2025. Participants were divided chronologically into a training cohort and an independent temporal validation cohort. Women recruited from June 2024 to January 2025 were included in the training cohort (n = 794), whereas those recruited from February 2025 to May 2025 were included in the temporal validation cohort (n = 341). At six weeks postpartum, PP-PTSD symptoms were assessed using the Post-traumatic Stress Disorder Checklist-Civilian Version (PCL-C), with a score ≥38 indicating probable PP-PTSD. Multidimensional variables, including physiological and psychological factors, environmental and family-related factors, and social-behavioral factors, were collected. Candidate predictors were first screened using univariate analysis and then selected using least absolute shrinkage and selection operator (LASSO) regression. Multivariable logistic regression was used to identify independent associated factors. Seven machine learning models, including Logistic Regression, Naive Bayes, Support Vector Machine, Decision Tree, Gradient Boosting, AdaBoost, and Linear Discriminant Analysis, were constructed. Model performance was evaluated in the independent temporal validation cohort using receiver operating characteristic curves, calibration curves, decision curve analysis, and the DeLong test. SHAP analysis was used to interpret the optimal model.

**Results:**

Among the 794 participants in the training cohort, the incidence of PP-PTSD was 25.18%. Five key predictors were selected by LASSO regression: social support, depression, neonatal caregiving style, husband’s participation, and sleep quality. Multivariable logistic regression showed that depression and poor sleep quality were associated with an increased risk of PP-PTSD, whereas higher social support, greater husband’s participation, and parental assistance in neonatal care were associated with a reduced risk. Among the seven models, the Gradient Boosting model achieved the best overall performance in the temporal validation cohort, with an AUC of 0.939, F1 score of 0.700, specificity of 0.943, sensitivity of 0.651, and Youden index of 0.595. The DeLong test showed that Gradient Boosting performed significantly better than Logistic Regression. SHAP analysis further indicated that social support, husband’s participation, sleep quality, and depression were the major contributors to model prediction.

**Conclusion:**

Postpartum PTSD (PP-PTSD) exhibits a higher incidence among primiparous women and exerts substantial adverse effects on maternal mental health, the mother–infant relationship, and overall family functioning. Guided by the Integrated Framework of Perinatal Health Risk Management (IFPHRM), this study elucidated the multidimensional mechanisms underlying PP-PTSD, encompassing physiological and psychological factors (e.g., sleep quality and depression), environmental and occupational factors (e.g., social support, paternal involvement, and infant caregiving practices), and social behavioral factors. The Gradient Boosting prediction model demonstrated robust performance and high predictive accuracy upon independent external validation, highlighting its potential utility for risk stratification and future clinical translation. Nevertheless, multicentre validation and the development of clinically implementable tools are warranted. Collectively, this study offers a theoretical foundation and methodological framework for the early identification, targeted intervention, and long-term health management of PP-PTSD in primiparous women.

## 1 Introduction

According to the World Health Organization’s 2023 Global Health Report, approximately 135 million women give birth worldwide each year, with the global prevalence of perinatal mental disorders ranging from 17.2% to 24.7% (1). This translates to roughly 40 million women experiencing psychological problems associated with childbirth annually. Maternal mental health has thus emerged as a critical global public health priority. Childbirth is generally regarded as a natural biological instinct for women and a joyful event that usually evokes positive psychological responses. However, this perception tends to overlook the fact that the uncertainty and unpredictability of the childbirth process may cause profound and lasting psychological harm to mothers. Studies have shown that traumatic experiences caused by adverse childbirth processes (2), fluctuations in hormone levels before and after delivery, as well as conflicts in maternal role adaptation and lifestyle changes, may all lead to psychological maladjustment and negative emotional responses in mothers. As a unique psychological experience for women, childbirth trauma has been reported to cause psychological distress in 9% to 44% of women, and in more severe cases may develop into postpartum post-traumatic stress disorder (PP-PTSD).

Post-traumatic stress disorder (PTSD) refers to delayed and persistent physical and psychological disturbances caused by exposure to extraordinarily threatening or catastrophic traumatic events (3). PP-PTSD refers to a delayed mental stress disorder occurring after childbirth, which may be triggered by previous traumatic experiences, pregnancy and delivery complications, traumatic childbirth experiences, or the birth of critically ill infants (4). Its main symptoms include re-experiencing negative feelings related to childbirth, avoidance of anything associated with delivery or the infant, heightened vigilance, and a state of excessive stress. PP-PTSD can seriously affect women’s physical and mental health, family relationships, and social relationships, and its impact may persist for a long time. Women with PP-PTSD are more likely to experience irritability, sadness, depression, sleep disturbances, and even suicidal ideation. The prevalence of PP-PTSD varies considerably across countries and regions. A cross-sectional study in Spain that assessed 290 postpartum women at 4–6 weeks after childbirth found a PP-PTSD prevalence of 10.6% (5). A multicenter study conducted by Dutch scholars in midwifery institutions, general hospitals, and teaching hospitals reported a PP-PTSD prevalence of 1.2% (6). In China, Xu Guiru et al. surveyed 233 postpartum women and reported a PP-PTSD prevalence of 10.3% (7). Among maternal populations, primiparas represent an important group in women’s health research because they undergo unique physiological, psychological, and social changes. Primiparas not only face the challenges of pregnancy, childbirth, and postpartum recovery, but also need to adapt to the transition to motherhood (8). Owing to their lack of prenatal and postnatal experience, primiparas often experience greater anxiety and stress, making them more vulnerable to PP-PTSD. Seng JS and colleagues in the United States followed 1, 581 primiparas (6) and found that the prevalence of PP-PTSD was 32%, which was much higher than that in multiparas.

Given the various risks associated with PP-PTSD, healthcare professionals must promptly understand the post-traumatic psychological responses of primiparas in order to implement appropriate short- and long-term medical interventions and alleviate problems caused by PP-PTSD symptoms. At present, research on PP-PTSD mainly focuses on investigations of its current status and influencing factors. Studies on influencing factors have primarily explored psychosocial-related variables (9, 10). However, comprehensive assessment of PP-PTSD among primiparas remains inadequate, and there is still a lack of studies exploring influencing factors from an overall perspective and constructing predictive models.

However, comprehensive assessment of PP-PTSD among primiparas remains inadequate, and there is still a lack of studies exploring influencing factors from an overall perspective and constructing predictive models (11) and has been widely used for the systematic and comprehensive assessment of various health problems, particularly in the fields of healthcare and environmental health. Its core components include health management and risk management. Health management aims to promote positive health determinants through regulation, community action, technical support, economic incentives, and counseling services. Risk management, by contrast, advocates the avoidance of negative health determinants and mainly includes physiological and psychological factors, environmental and occupational factors, as well as social and behavioral factors. Meanwhile, machine learning (ML) has developed into a powerful computer-assisted method for data mining and analysis (12, 13) and has been widely applied as a predictive tool in various engineering and medical fields. Its predictive accuracy is superior to that of traditional statistical methods (14). Therefore, based on the IFPHRM, this study aimed to explore the potential physiological, genetic, psychological, environmental, occupational, social, and behavioral factors influencing primiparas, while also developing an integrated predictive model based on multiple ML algorithms (including logistic regression, random forest, and XGBoost), so as to provide evidence for identifying high-risk populations and implementing preventive measures.

## 2 Materials and methods

### 2.1 Study participants

This cross-sectional study was conducted between June 2024 and May 2025 in the Department of Obstetrics at Hefei Maternal and Child Health Hospital. Primiparous women who delivered during this period were consecutively recruited. Participants were assessed at 6 weeks postpartum using a validated Posttraumatic Stress Disorder Scale. PP-PTSD was determined based on the established cutoff scores. Unlike random allocation, a temporal split was employed: participants enrolled from June 2024 to January 2025 (n=794) constituted the training cohort, while those enrolled from February 2025 to May 2025 (n=341) formed the external validation cohort. This design was intentionally chosen to mimic real-world clinical prediction settings and thereby enhance the model’s generalizability and practical utility. To prevent data leakage, all feature selection procedures—including univariate analysis and LASSO regression—as well as model training and hyperparameter tuning were performed exclusively on the training cohort. The external validation cohort was reserved solely for final performance evaluation and was not involved in any variable selection or model optimization steps. The study protocol was approved by the Ethics Committee of Anhui Medical University Affiliated Hefei Maternal and Child Health Care Hospital, and written informed consent was obtained from all participants.

### 2.2 Inclusion and exclusion criteria

The inclusion criteria were as follows: (1) primiparous women with singleton delivery; (2) age ≥18 years; (3) delivery at the study hospital with planned completion of the 6-week postpartum follow-up; (4) ability to read and communicate, and ability to complete the questionnaire independently or with guidance from the researchers; and (5) voluntary participation with signed informed consent.

The exclusion criteria were as follows: (1) a prior confirmed diagnosis of severe mental disorders or current receipt of systematic antipsychotic treatment, such as schizophrenia or bipolar disorder; (2) a prior confirmed diagnosis of PTSD or severe depression or anxiety requiring long-term medication; and (3) severe physical illness or obstetric critical conditions that prevented completion of the follow-up assessment.

### 2.3 Methods

#### 2.3.1 Predictors

This study was based on the Integrated Framework for Population Health Risk Management (IFPHRM) and examined predictors from three dimensions: physiological and psychological factors, environmental and occupational factors, and social and behavioral factors. Combined with a literature review, predictors were screened and a total of 40 related factors were identified. Specifically, these included: (1) physiological and psychological factors, including age, BMI, sleep quality, and anxiety-depressive symptoms; (2) environmental and occupational factors, including residential area, educational level, average monthly household income, neonatal health status, neonatal caregiving style, and husband’s participation; and (3) social and behavioral factors, including social support, mode of delivery, delivery status.

#### 2.3.2 Instruments

##### 2.3.2.1 Post-traumatic stress disorder

Post-traumatic stress disorder was assessed using the Post-traumatic Stress Disorder Checklist-Civilian Version (PCL-C) (15). The scale includes three dimensions: re-experiencing symptoms, avoidance symptoms, and hyperarousal symptoms, with a total of 17 items. A 5-point Likert scale was used, with a maximum score of 85. A PCL-C score ≥38 was considered indicative of PTSD, and higher scores indicated more severe postpartum PTSD symptoms. In this study, the Cronbach’s α coefficient of the scale was 0.849, indicating good internal consistency.

##### 2.3.2.2 Sleep quality

Sleep quality was assessed using the Pittsburgh Sleep Quality Index (PSQI) (16). The scale includes seven dimensions: subjective sleep quality, sleep latency, sleep duration, habitual sleep efficiency, sleep disturbances, use of sleeping medication, and daytime dysfunction, comprising a total of 18 items. The total score ranges from 0 to 21, with higher scores indicating poorer sleep quality. In this study, the Cronbach’s α coefficient of the scale was 0.853, indicating good internal consistency.

##### 2.3.2.3 Anxiety and depressive symptoms

Anxiety and depressive symptoms were assessed using the Hospital Anxiety and Depression Scale (HADS), originally developed by Zigmond and Snaith in 1983 and later translated and revised into Chinese by Ye Weifei et al. (17). The scale consists of 14 items, with the odd-numbered items forming the anxiety subscale and the even-numbered items forming the depression subscale. A 4-point Likert scale was used, with each item scored from 0 to 3, and higher scores indicating more severe symptoms. According to the scoring criteria, a score of >7 indicates the presence of anxiety or depressive symptoms. In this study, the Cronbach’s α coefficient of the scale was 0.892, indicating good internal consistency.

##### 2.3.2.4 Social support

Social support was assessed using the Social Support Rating Scale (SSRS) (18). The scale includes three dimensions: subjective support, objective support, and utilization of support, with a total of 10 items and a maximum score of 66. Higher scores indicate a higher level of social support. A score of <20 indicates a low level of social support, a score of 20–30 indicates a moderate level, and a score of >30 indicates a high level of social support. The Cronbach’s α coefficient of the scale was 0.862, reflecting good internal consistency in measuring social support.

##### 2.3.2.5 General information questionnaire

The questionnaire was developed in consultation with neurologists, nursing specialists, and biostatisticians. Guided by the Integrated Framework for Population Health Risk Management (IFPHRM), it encompassed variables across three domains: (1) physiological and psychological factors (maternal age and body mass index [BMI]); (2) environmental and occupational factors, including residential area, maternal education level, average monthly family income, neonatal health status (abnormal: Apgar score <7 at birth), neonatal caregiving practices, and husband’s participation in infant care; and (3) socio-behavioral factors, including mode of delivery, gestational age at birth (preterm: <37 weeks; term: 37–42 weeks; post-term: >42 weeks), and labor progress (abnormal versus normal). Abnormal labor was defined, according to clinical records, as a prolonged first stage (>12 hours), prolonged second stage (>2 hours), prolonged third stage (>30 minutes), or the need for instrumental assistance (forceps or vacuum extraction).

### 2.4 Statistical analysis

All statistical analyses and model development were performed using SPSS version 26.0, R version 4.5.2, and Python version 3.12.7. Categorical variables are presented as counts (percentages), and intergroup comparisons were conducted using the χ² test. Normally distributed continuous variables were compared using the independent-samples t-test. Variables showing P < 0.05 in univariate analysis were entered into LASSO regression (conducted exclusively in the training set, n = 794) for feature selection, thereby reducing redundancy in high-dimensional data and mitigating overfitting risk. Multicollinearity among selected predictors was assessed using variance inflation factor (VIF). Retained variables were subsequently included in multivariable logistic regression models. Prior to modeling, data completeness was evaluated: continuous variables with <5% missing values were imputed using multiple imputation by chained equations (MICE); categorical variables with missing data were imputed using the mode; variables with >20% missingness were excluded to ensure model reliability and result reproducibility. Class imbalance was addressed using the Synthetic Minority Over-sampling Technique (SMOTE). Model stability was evaluated through 1, 000 bootstrap resamples to compute 95% confidence intervals for the area under the receiver operating characteristic curve (AUC) and to test for significant differences between models. Calibration curves and decision curve analysis (DCA) were applied to assess discrimination, calibration, and net clinical benefit, respectively. The DeLong test was used to compare AUCs across models. The optimal model was further interpreted using SHAP (SHapley Additive exPlanations) values, with corresponding swarm plots generated. All statistical tests were two-sided, and a P-value < 0.05 was considered statistically significant.

## 3 Results

### 3.1 Univariate analysis

The results of the univariate analysis (**Table 1**) showed that, in the training set, there were statistically significant differences between the PP-PTSD group and the non-PP-PTSD group in depression, level of social support, neonatal caregiving style, husband’s participation, educational level, neonatal health status, C-reactive protein, anxiety, sleep quality, and age (all P < 0.05). In contrast, no significant between-group differences were found in BMI, monthly household income, delivery status (preterm/full-term/post-term), residential area, labor status, or mode of delivery (all P > 0.05).

**Table 1:** Univariate analysis of postpartum post-traumatic stress disorder in primiparas.

Variable	Category	Normal (n = 594)	PP-PTSD (n = 200)	Statistic	P value
Depression	No	443 (74.58%)	81 (40.50%)	χ²=75.921	<0.001
	Yes	151 (25.42%)	119 (59.50%)		
Social support	Low	78 (13.13%)	101 (50.50%)	χ²=172.222	<0.001
	Moderate	136 (22.90%)	70 (35.00%)		
	High	380 (63.97%)	29 (14.50%)		
Neonatal caregiving style	Self-care	191 (32.15%)	115 (57.50%)	χ²=39.514	<0.001
	Parental assistance	403 (67.85%)	85 (42.50%)		
Husband’s participation	Low	65 (10.94%)	101 (50.50%)	χ²=181.651	<0.001
	Moderate	142 (23.91%)	65 (32.50%)		
	High	387 (65.15%)	34 (17.00%)		
BMI	≤24kg/m^2^	251 (42.26%)	99 (49.50%)	χ²=2.898	0.0887
	>24kg/m^2^	343 (57.74%)	101 (50.50%)		
Monthly income	<2000	244 (41.08%)	88 (44.00%)	χ²=4.115	0.1278
	2000˜5000	225 (37.88%)	83 (41.50%)		
	>5000	125 (21.04%)	29 (14.50%)		
Educational level	Illiterate	127 (21.38%)	92 (46.00%)	χ²=45.761	<0.001
	Primary school or below	153 (25.76%)	39 (19.50%)		
	Junior high school or above	314 (52.86%)	69 (34.50%)		
Delivery status	Preterm	166 (27.95%)	51 (25.50%)	χ²=1.241	0.5376
	Full-term	323 (54.38%)	107 (53.50%)		
	Post-term	105 (17.68%)	42 (21.00%)		
Residential area	Rural	329 (55.39%)	114 (57.00%)	χ²=0.099	0.7528
	Urban	265 (44.61%)	86 (43.00%)		
Labor status	Abnormal	140 (23.57%)	42 (21.00%)	χ²=0.423	0.5155
	Normal	454 (76.43%)	158 (79.00%)		
Neonatal health status	Normal	535 (90.07%)	147 (73.50%)	χ²=32.542	<0.001
	Abnormal	59 (9.93%)	53 (26.50%)		
Mode of delivery	Vaginal delivery	417 (70.20%)	141 (70.50%)	χ²=0.001	0.999
	Cesarean section	177 (29.80%)	59 (29.50%)		
C-reactive protein	Normal	509 (85.69%)	136 (68.00%)	χ²=29.566	<0.001
	Abnormal	85 (14.31%)	64 (32.00%)		
Anxiety	No	502 (84.51%)	154 (77.00%)	χ²=5.368	0.0205
	Yes	92 (15.49%)	46 (23.00%)		
Sleep quality^a^	—	10.0 (Q1: 7.0, Q3: 13.0)	14.0 (Q1: 11.0, Q3: 17.0)	U=27230.0	<0.001
Age^a^	—	27.0(Q1:25.0, Q3: 30.0)	26.0 (Q1: 24.0, Q3: 29.0)	U=73439.0	<0.001

* Data are presented as n (%) or median (Q1, Q3). Superscript ‘a’ denotes continuous variables (sleep quality, measured by PSQI score; age in years). PP-PTSD, postpartum post-traumatic stress disorder.

### 3.2 Variable selection

**Figure 1:**
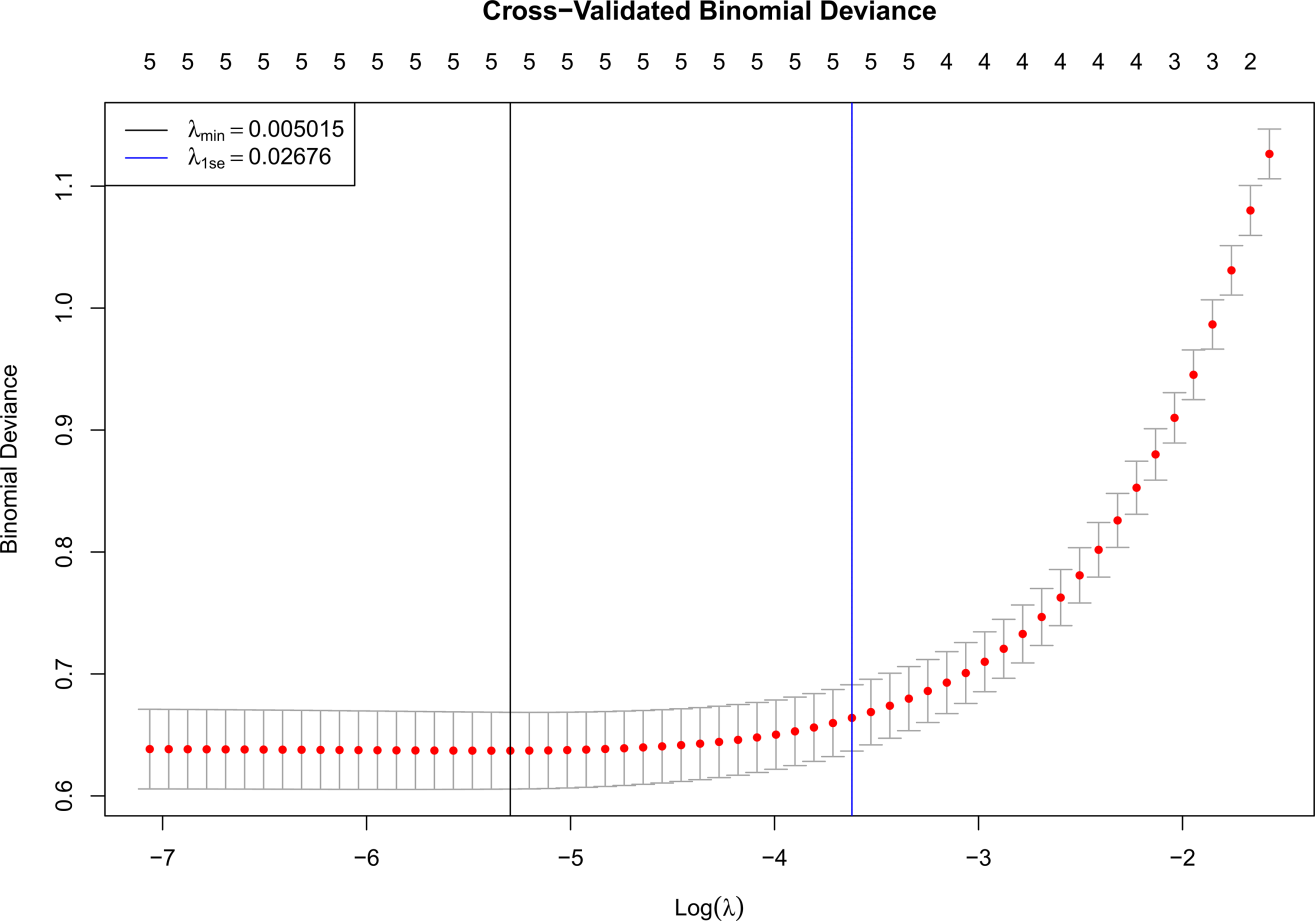
Feature selection for postpartum post-traumatic stress disorder in primiparas using LASSO regression.

Variables with statistically significant differences in the univariate analysis (P < 0.05) were further subjected to LASSO regression for feature selection (**Figure 1**). Through cross-validation performed on the training set, five core predictors with non-zero coefficients were ultimately retained: social support level, depression, infant care mode, husband involvement, and sleep quality. Subsequently, multicollinearity diagnostics were performed on the selected variables. The variance inflation factor (VIF) for each variable was below 5, indicating no significant multicollinearity. Thus, these variables were suitable for subsequent multivariate model building and predictive model development.

### 3.3 Multivariable analysis

The five predictors selected by LASSO were included in the multivariable logistic regression analysis. As shown in **Table 2**, using low social support as the reference category, high social support was significantly associated with a reduced risk of PP-PTSD (OR = 0.103, 95% CI: 0.056–0.191), whereas moderate social support was not significantly associated with PP-PTSD (OR = 0.941, 95% CI: 0.541–1.637). Using no depression as the reference category, depression was significantly associated with an increased risk of PP-PTSD (OR = 5.667, 95% CI: 3.423–9.384). Using self-care as the reference category, parental assistance in neonatal care was associated with a lower risk of PP-PTSD (OR = 0.502, 95% CI: 0.300–0.840). Using low husband’s participation as the reference category, both moderate participation (OR = 0.523, 95% CI: 0.294–0.931) and high participation (OR = 0.059, 95% CI: 0.032–0.112) were associated with a reduced risk of PP-PTSD. In addition, higher sleep quality scores were significantly associated with an increased risk of PP-PTSD (OR = 2.171, 95% CI: 1.702–2.770).

**Table 2:** Multivariable logistic regression analysis of postpartum post-traumatic stress disorder in primiparas.

Predictors	β	SE	Odds Ratio	95%CI	P
Social support (reference: low)					
Moderate	-0.061	0.283	0.941	[0.541, 1.637]	0.829
High	-2.273	0.315	0.103	[0.056, 0.191]	<0.001
Depression (reference: no)					
Yes	1.735	0.257	5.667	[3.423, 9.384]	<0.001
Neonatal caregiving style (reference: self-care)					
Parental assistance	-0.689	0.262	0.502	[0.300, 0.840]	0.009
Husband’s participation (reference: low)					
Moderate	-0.647	0.294	0.523	[0.294, 0.931]	0.028
High	-2.822	0.322	0.059	[0.032, 0.112]	<0.001
Sleep quality					
	0.775	0.124	2.171	[1.702, 2.770]	<0.001

### 3.4 Model construction and validation

**Figure 2:**
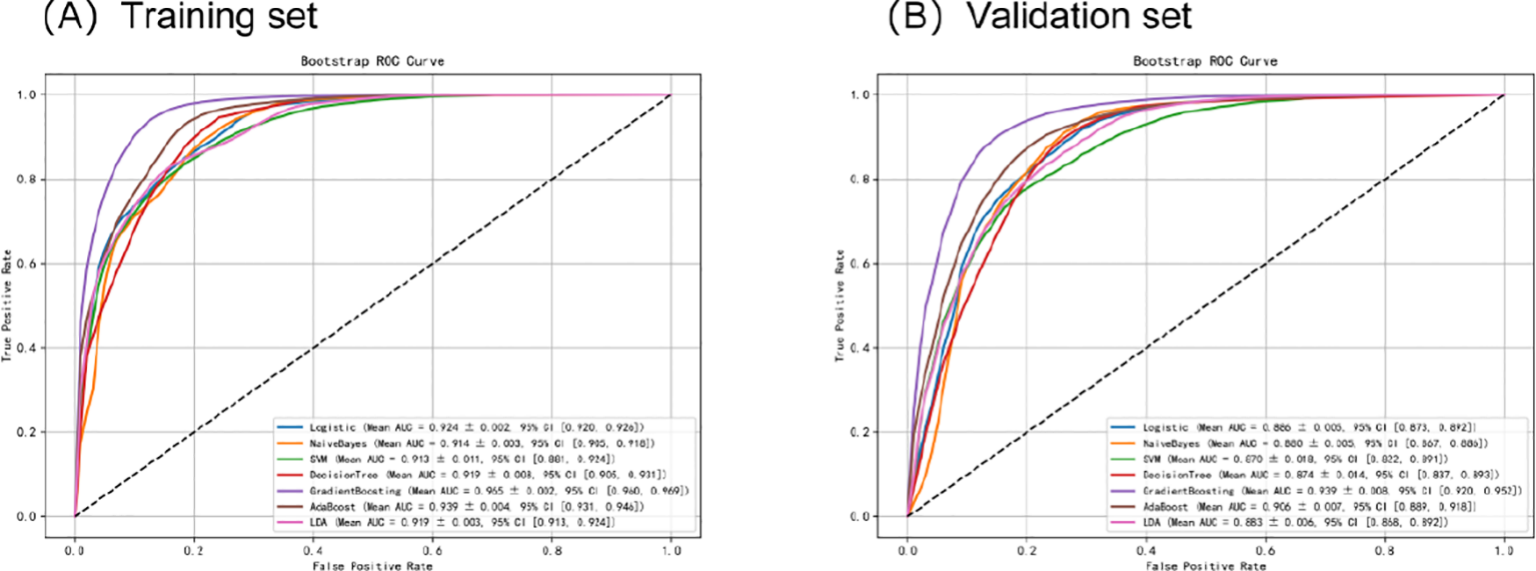
ROC curves of risk prediction models for postpartum post-traumatic stress disorder in primiparas.

**Figure 3:**
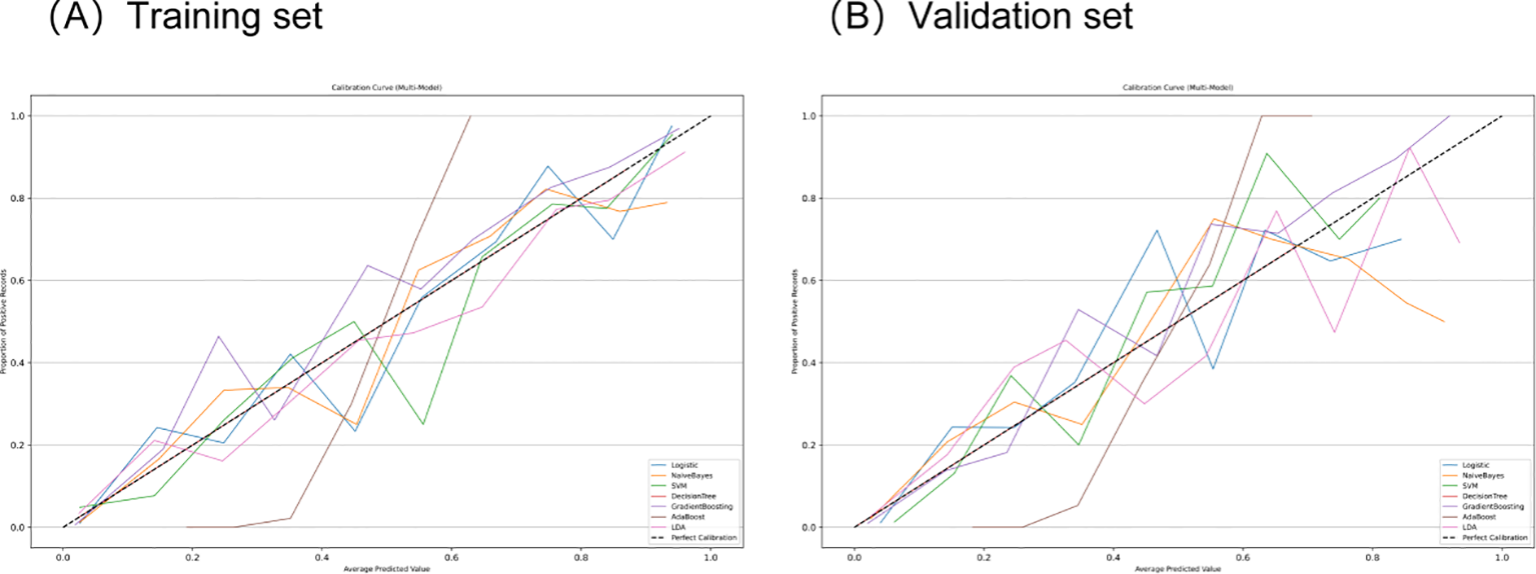
Decision curve analysis (DCA) curves of risk prediction models for postpartum post-traumatic stress disorder in primiparas.

**Figure 4:**
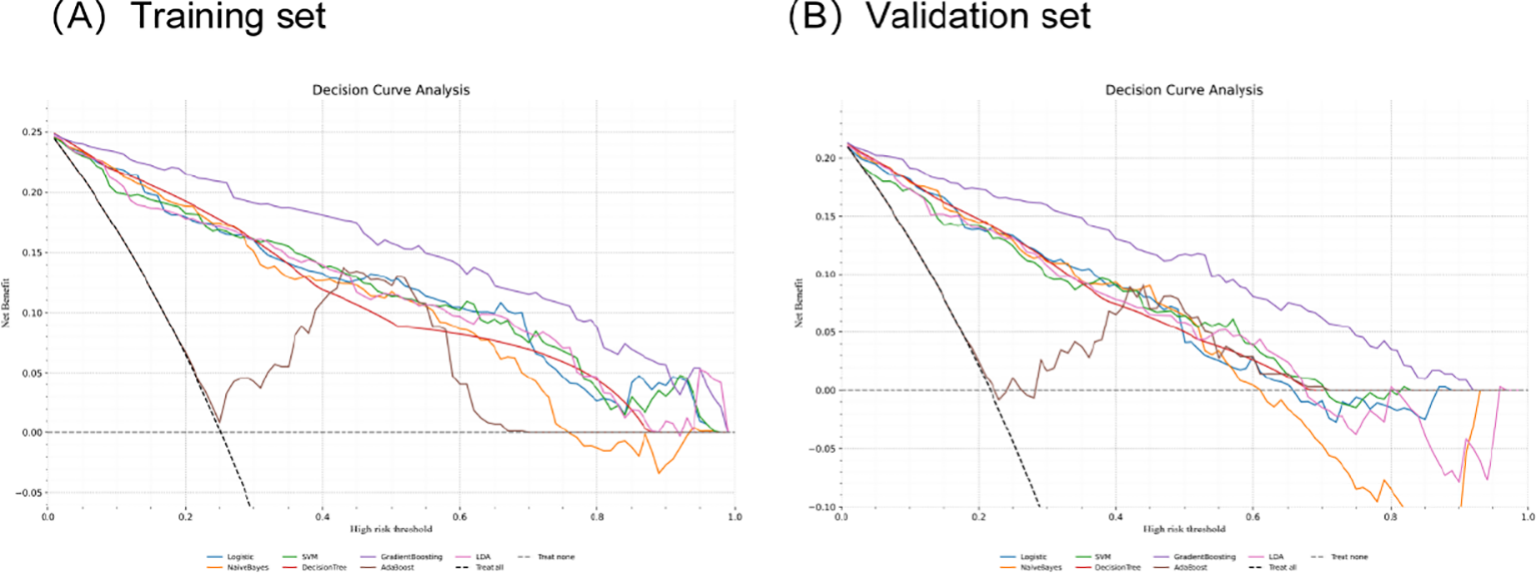
Calibration curves of risk prediction models for postpartum post-traumatic stress disorder in primiparas.

In this study, multiple models, including Logistic Regression, Naive Bayes, SVM, Decision Tree, Gradient Boosting, AdaBoost, and LDA, were compared. ROC curves (**Figure 2**), calibration curves (**Figure 3**), and decision curves were plotted for both the training and validation sets to provide a comprehensive evaluation (**Figure 4**). The ROC results showed that the curves of the different models were generally close to each other, with AUCs in the training set generally higher than those in the validation set. The AUCs in the validation set were mostly around 0.8, indicating good discrimination with no substantial overall differences among the models. The calibration curves showed that most models were generally close to the ideal calibration line, suggesting acceptable agreement between predicted probabilities and observed outcomes, although some deviation remained in certain probability ranges. The DCA results indicated that, across a relatively wide range of threshold probabilities, most models achieved positive net benefit, suggesting that these models have potential clinical value for risk stratification of PP-PTSD in primiparas.

### 3.5 Comparison of multiple models

This study developed a predictive model for PP-PTSD using seven machine learning algorithms and performed a comprehensive evaluation on the external validation set using ROC curves, calibration curves, and decision curve analysis (DCA). The calibration curve showed that the model’s predictions aligned closely with the ideal calibration line in the low-to-medium risk probability range, but deviated slightly in the high-probability range, suggesting that the model should be interpreted with caution when predicting extremely high risk. Decision curve analysis (DCA) for the validation set further indicated that within the threshold probability range of 0.2–0.6, the Gradient Boosting, AdaBoost, and Logistic regression models all yielded positive net benefits, demonstrating their clinical utility for early risk screening. Among all models, Gradient Boosting exhibited the best overall performance, achieving an AUC of 0.939, an F1 score of 0.700, a specificity of 0.943, a sensitivity of 0.651, a Youden index of 0.595. These metrics indicate that Gradient Boosting offers superior detection capability while maintaining high specificity, and that its predicted probabilities had the best agreement with actual outcomes. The difference between Gradient Boosting and the reference Logistic regression model was statistically significant (P = 0.001). In contrast, AdaBoost achieved a relatively high AUC (0.906) but showed only moderate F1 and sensitivity. Decision Tree exhibited the weakest overall performance. The remaining models (LDA, Naive Bayes, SVM) had performance metrics falling between those of Gradient Boosting and Logistic regression, and showed no statistically significant difference from Logistic regression (**Table 3**).

**Table 3:** Comparison of multiple machine learning models for predicting postpartum post-traumatic stress disorder in primiparas.

Model name	AUC(95% CI)	F1	Specificity	Sensitivity	Youden index	P value
AdaBoost	0.906((0.874–0.938))	0.623	0.928	0.573	0.500	0.037
Decision Tree	0.874(0.837–0.911)	0.546	0.903	0.526	0.429	0.694
Gradient Boosting	0.939(0.911–0.967)	0.700	0.943	0.651	0.595	0.001
LDA	0.883(0.848–0.918)	0.604	0.908	0.579	0.487	0.485
Logistic	0.886(0.851–0.921)	0.577	0.920	0.526	0.447	Reference
Naïve Bayes	0.880(0.844–0.916)	0.610	0.897	0.604	0.501	0.162
SVM	0.870(0.832–0.908)	0.587	0.913	0.553	0.466	0.343

*P values were calculated using the DeLong test for pairwise comparison of AUCs, with Logistic Regression used as the reference model.

### 3.6 Ranking of influencing factors based on SHAP analysis

**Figure 5:**
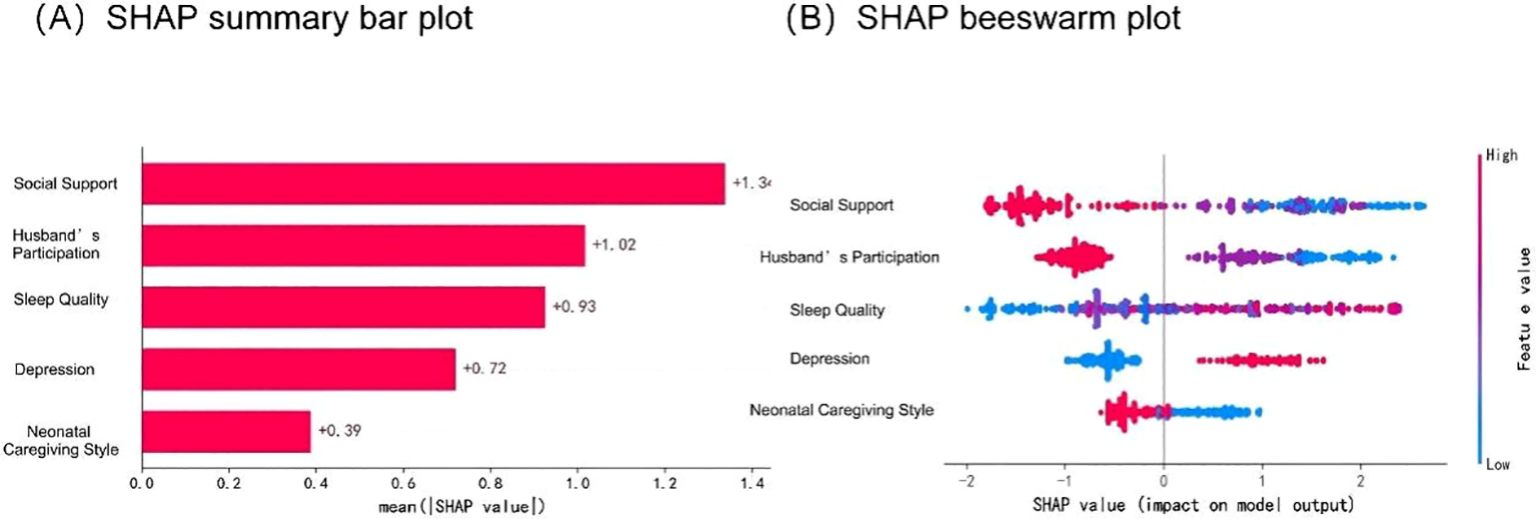
SHAP analysis of the gradient boosting risk prediction model for postpartum post-traumatic stress disorder in primiparas.

SHAP analysis revealed that, among all features, social support level had the highest mean absolute SHAP value (**Figures 5A, B**), indicating its dominant contribution to model predictions. High social support yielded a markedly negative SHAP value, which lowered the predicted probability, confirming it as the strongest protective factor. Clinically, this finding suggests that early postpartum support—through family, community, and professional networks—can significantly buffer traumatic stress. Husband involvement was identified as the second most important protective factor. High involvement generated a strong negative SHAP value, whereas low involvement produced a positive contribution. Clinically, this underscores that encouraging husbands to actively participate in newborn care and emotional support represents a low-cost, high-efficiency intervention target. Regarding sleep quality, a higher PSQI score (indicating poorer sleep) produced a positive SHAP value, establishing it as an important risk factor. Similarly, individuals with depressive symptoms predominantly yielded positive SHAP values, identifying them as strong risk factors. Additionally, regarding infant care mode, care provided by grandparents (as opposed to self-care) produced a negative SHAP contribution, although its effect size was smaller than that of the factors mentioned above.

The SHAP beeswarm plot further revealed a directional distribution of feature values at the sample level: high risk feature values clustered predominantly in the positive SHAP region, driving the model’s prediction toward PP-PTSD, whereas protective feature values were more dispersed in the negative SHAP region. This pattern indicates that the model’s decisions align well with the risk directions identified in the multivariable analysis, demonstrating good interpretability and stability.

## 4 Discussion

### 4.1 Current status of PP-PTSD incidence

Postpartum post-traumatic stress disorder is a common psychological disorder among primiparas after childbirth. Previous studies in China and abroad have reported an incidence of approximately 12.24%, which may exceed 25% in some regions or specific populations (19). In the present study, the incidence was 25.18%. PP-PTSD not only seriously impairs maternal psychological and physical health and may lead to a series of problems such as sleep disturbances and comorbid depression, but may also disrupt the mother–infant attachment relationship, impair family functioning, and exert long-term adverse effects on the emotional regulation and development of newborns (20).

### 4.2 Influencing factors of PP-PTSD

#### 4.2.1 Physiological and psychological factors

This study showed that sleep quality significantly affected the risk of PP-PTSD (OR = 2.171, 95% CI: 1.702–2.770). Sleep disturbance is a common postpartum problem, especially in the early postpartum period, when frequent nighttime infant feeding and physiological hormonal changes often lead to decreased sleep quality. Sleep quality directly affects brain recovery and stress responses (21). Previous studies have indicated that poor sleep quality is closely associated with impaired emotional regulation (22), which may increase sensitivity to negative emotions and intensify the intrusiveness of traumatic memories. Poor-quality sleep not only affects emotional regulation, but also interferes with cognitive function, resulting in reduced adaptability to postpartum stress and thereby increasing the risk of PTSD (23). Therefore, as a physiological factor, sleep quality plays a significant role in PP-PTSD, suggesting that sleep intervention may be a key strategy for the prevention and treatment of PP-PTSD.

The relationship between depressive symptoms and PP-PTSD is particularly close. In this study, depressive symptoms were identified as one of the strongest risk factors (OR = 5.667). Depression may increase the risk of post-traumatic stress responses through several mechanisms (24–26). First, individuals with depression often exhibit strong negative cognitive tendencies, such as catastrophizing and helplessness, which can aggravate negative postpartum emotions. Second, depression may reduce an individual’s coping resources, leaving them without adequate emotional regulation and coping capacity when facing traumatic events, thereby worsening trauma-related responses. Therefore, depressive symptoms have a significant impact on the occurrence of postpartum trauma, highlighting the importance of psychological intervention.

Of note, the odds ratios for sleep quality and depression observed in this study reflect measures of association rather than causal effects. Poor sleep and depression may serve both as risk factors for PP-PTSD and as its symptoms or comorbid outcomes. A bidirectional relationship may exist among these three factors; nonetheless, sleep quality and depressive status remain effective markers for risk stratification of PP-PTSD (27). This cycle of “sleep–emotion–stress” suggests that interventions should combine the treatment of depression with improvement in sleep quality to help mothers break this vicious cycle and reduce the occurrence of PP-PTSD.

#### 4.2.2 Environmental and occupational factors

Husband’s participation was identified as key factor (OR = 0.059), and its role should not be overlooked. Previous studies have shown that high husband participation can effectively reduce the psychological burden on mothers and promote emotional recovery (28). Husband’s participation is not limited to practical assistance, such as caring for the newborn and sharing household responsibilities, but also includes emotional support, listening, and encouragement. Active husband participation provides mothers with greater emotional security and effectively alleviates childcare-related stress. Compared with low husband participation, high participation can better help mothers adjust psychologically, reduce anxiety and depressive symptoms, and lower the risk of PP-PTSD (29, 30). Therefore, the role of husbands in the postpartum family support system should not be underestimated. Although high husband involvement was strongly correlated with a low risk of PP-PTSD, the cross-sectional design of this study cannot rule out the possibility of reverse causation. This study posits that mothers with more severe PP-PTSD symptoms may, as a result of avoidance behaviors or emotional changes, actively reduce their perception or acceptance of their husband’s involvement. Therefore, the clinical utility of assessing husband involvement lies primarily in screening for low involvement among mothers at low risk of PP-PTSD.

Neonatal caregiving style was also included in the final LASSO-selected model (OR = 0.502). This study showed that, compared with self-care, parental assistance significantly reduced the risk of PP-PTSD in mothers. This finding suggests that intergenerational support has an important influence on maternal mental health (31). Assistance from parents not only reduces childcare burden, but also decreases mothers’ helplessness and anxiety when caring for the newborn by providing parenting experience and guidance (32). Especially for primiparas, parental caregiving offers not only physical support, but also emotional comfort and psychological stability. Therefore, appropriate family support can not only reduce maternal stress, but also effectively promote psychological recovery. Furthermore, social support exhibited a significant negative correlation with the risk of PP-PTSD in this study, highlighting its protective effect on maternal mental health.

#### 4.2.3 Social and behavioral factors

In this study, social support was identified as one of the most important protective factors and showed a strong protective effect in the LASSO-selected model (OR = 0.103). A high level of social support was significantly associated with a reduced risk of PP-PTSD. This finding is consistent with previous studies (33). Social support helps mothers cope with postpartum stress and enhances their recovery capacity through emotional support, informational support, and instrumental support (34). Emotional support mainly helps relieve anxiety and depressive symptoms by reducing feelings of loneliness and enhancing emotional security, whereas informational support helps mothers better understand the processes of infant care and physical recovery, thereby reducing anxiety caused by uncertainty (35). The strength of social support directly affects maternal recovery from and adaptation to post-traumatic symptoms (36). This suggests that enhancing social support may indirectly prevent PP-PTSD by alleviating the mother’s psychological burden. From a clinical standpoint, strengthening social support networks—particularly during the early postpartum period—through the active engagement of family members, friends, or professional psychological services can provide mothers with more robust emotional and practical support, thereby reducing their stress response and post-traumatic symptoms, and consequently lowering the risk of PP-PTSD. Future intervention strategies should focus on improving maternal social support levels and enhancing emotional resilience through community- and family-based support. Therefore, a robust social support network is crucial for preventing PP-PTSD.

This study showed that sleep quality and emotional status are important physiological and psychological mechanisms underlying the development of PP-PTSD. This suggests that postpartum psychological interventions should pay close attention to sleep problems and the training of emotional regulation. Behavioral interventions, such as cognitive behavioral therapy (CBT) (37) and mindfulness-based stress reduction (MBSR) (38), have been shown to be effective in improving postpartum depression, anxiety, and sleep quality. Therefore, developing comprehensive interventions that target sleep quality and emotional status may be an effective strategy for the prevention and treatment of PP-PTSD. This study also emphasized the importance of social support, indicating that postpartum emotional and social support networks play a key role in preventing PP-PTSD. In the future, social support services for mothers should be strengthened, not only through traditional family support, but also through more comprehensive support systems such as community support, psychological counseling services, and mother–infant interaction support. As part of the support system, the active involvement of family members, especially husbands, plays an important role in reducing the psychological burden on mothers.

This study also highlighted the influence of neonatal caregiving style on maternal mental health, particularly the involvement of parents. On this basis, policymakers and society should promote more inclusive and supportive parenting policies, encouraging family members to participate jointly in childcare and reducing the unilateral burden on mothers. Particularly among primiparas, the development of family support systems is especially important and can be further strengthened through measures such as parenting guidance and parent–child relationship building programs.

### 4.3 Construction and validation of multiple machine learning models for PP-PTSD prediction

In this study, PP-PTSD prediction models were developed and compared using multiple machine learning methods, including Logistic Regression, Naive Bayes, SVM, Decision Tree, Gradient Boosting, AdaBoost, and LDA. Comprehensive evaluation was performed in both the training and validation sets using ROC curves, calibration curves, and decision curve analysis (DCA). The results showed that all models achieved AUCs of approximately 0.8 in the validation set, indicating good discriminative ability. The calibration curves were generally close to the ideal line, suggesting good agreement between predicted probabilities and observed outcomes. DCA further demonstrated that all models achieved positive net benefit over a relatively wide range of threshold probabilities, indicating potential clinical utility.

Among all models compared, Gradient Boosting achieved the best performance (AUC = 0.939, F1 = 0.700, specificity = 0.943, Youden index = 0.595), delivering high specificity while maintaining reasonable sensitivity. Overall, its performance was significantly superior to that of the Logistic regression model (P = 0.001). Although AdaBoost yielded a higher AUC, its sensitivity was relatively modest; Decision Tree showed the weakest overall performance. The remaining models (LDA, Naive Bayes, SVM) showed no statistically significant difference in performance compared with the Logistic regression model. SHAP analysis further improved model interpretability: it not only validated the findings from traditional statistical analyses but also provided visual explanations of individual-level feature contributions. The direction of the SHAP effects aligned with the results of the multivariable Logistic analysis, thereby enhancing model stability and clinical credibility. The SHAP contributions identifying social support and husband involvement as the strongest protective factors suggest that clinical practice should establish a “family-community” integrated support model. This includes routinely assessing social support levels during postpartum follow-up and encouraging husbands to participate in parenting training. The positive risk contributions of sleep quality and depression emphasize that early postpartum screening using PSQI and HADS, combined with cognitive-behavioral therapy and sleep hygiene education, could effectively break this vicious cycle. The Gradient Boosting model demonstrated the best predictive performance in this study, offering good clinical utility for risk stratification and showing promise for early clinical screening. The model’s output risk probability can serve as an auxiliary reference for postpartum psychological assessment, helping healthcare providers identify individuals at high risk for subclinical PP-PTSD and promptly deliver psychological intervention and support, thereby reducing the incidence of PP-PTSD.

## 5 Limitations

Although this study adopted a multidimensional analytical framework and machine learning methods, several limitations should be acknowledged. First, this was a cross-sectional study without long-term follow-up, making it impossible to establish causal relationships. Second, Although measures such as cross-validation were implemented during model development to mitigate overfitting, and an independent validation set was used for performance evaluation, the relatively high AUC in the training set may still reflect some degree of overfitting to the specific training sample. Therefore, future studies should further validate the model’s performance using larger, multi-center, and geographically diverse samples, along with clinical translation assessments. Finally, although the model showed good predictive performance statistically, its clinical translation still requires further validation and the development of practical application tools.

## 6 Conclusion

Based on the Integrated Framework for Population Health Risk Management (IFPHRM), this study identified key factors associated with PP-PTSD in primiparas from physiological, psychological, environmental, social, and behavioral dimensions. Depression and poor sleep quality were the major risk factors, whereas social support, husband’s participation, and parental assistance were important protective factors. The Gradient Boosting model demonstrated good predictive performance and interpretability, and may provide a useful reference for the early identification, risk stratification, and targeted intervention of PP-PTSD in primiparas.

## Data Availability

The raw data supporting the conclusions of this article will be made available by the authors, without undue reservation.
